# Significance of *KDM6A* mutation in bladder cancer immune escape

**DOI:** 10.1186/s12885-021-08372-9

**Published:** 2021-05-29

**Authors:** Xingxing Chen, Xuehua Lin, Guofu Pang, Jian Deng, Qun Xie, Zhengrong Zhang

**Affiliations:** Department of Urology, Zhuhai People’s Hospital, Zhuhai Hospital affiliated with Jinan University, Kangning Road No.79, in Zhuhai city, Guang Dong Province, Zhuhai, People’s Republic of China

**Keywords:** *KDM6A*, Bladder cancer, Mutation, Immune

## Abstract

**Background:**

Bladder cancer (BC) is the fourth most prevalent neoplasm in men and is associated with high tumour recurrence rates, leading to major treatment challenges. Lysine-specific demethylase 6A (*KDM6A*) is frequently mutated in several cancer types; however, its effects on tumour progression and clinical outcome in BC remain unclear. Here, we explored the potential role of *KDM6A* in regulating the antitumor immune response.

**Methods:**

We mined The Cancer Genome Atlas (TCGA) and International Cancer Genome Consortium (ICGC) databases for somatic mutation and clinical data in patients with BC.

**Results:**

We found frequent mutations in 12 genes in both cohorts, including *TP53, KDM6A, CSMD3, MUC16, STAG2, PIK3CA, ARID1A, RB1, EP300, ERBB2, ERBB3*, and *FGFR3*. The frequency o *KDM6A* mutations in the TCGA and ICGC datasets was 25.97 and 24.27%, respectively. In addition, *KDM6A* mutation was associated with a lower number of tumour-infiltrating immune cells (TIICs) and indicated a state of immune tolerance. *KDM6A* mutation was associated with lower *KDM6A* mRNA level compared with that in samples carrying the wild-type gene. Further, survival analysis showed that the prognosis of patients with low *KDM6A* expression was worse than that with high *KDM6A* expression. Using the CIBERSORT algorithm, Tumor Immune Estimation Resource site, and Gene Set Enrichment Analysis, we found that *KDM6A* mutation downregulated nine signalling pathways that participate in the immune system and attenuated the tumour immune response.

**Conclusion:**

Overall, we conclude that *KDM6A* mutation is frequent in BC and promotes tumour immune escape, which may serve as a novel biomarker to predict the immune response.

**Supplementary Information:**

The online version contains supplementary material available at 10.1186/s12885-021-08372-9.

## Introduction

Bladder cancer (BC) is considered to be the fourth most prevalent neoplasm in men [1]. BC is associated with high rates of tumour recurrence and progression, which represent major challenges in treatment. Lysine-specific demethylase 6A (*KDM6A*) is a member of the histone H3 lysine 27 demethylase gene family, which is reported to exert pro-tumorigenic effects in some cancer types [2, 3] but is also considered a tumour suppressor in other contexts [4]. Analyses of tumour-associated genes showed frequent loss of *KDM6A* expression in several cancers, including BC, B-cell lymphoma, lung squamous cell carcinoma, head and neck squamous cell carcinoma, pancreatic adenocarcinoma, and renal papillary cell carcinoma [5–9]. Nevertheless, the molecular mechanism by which *KDM6A* might suppress tumour progression in BC remains unclear. To provide insight into this mechanism, we conducted data mining of two separate public cohorts of BC patients to explore the frequency of *KDM6A* mutations and its potential influence on the immune response. This study can help to elucidate the effect of *KDM6A* on the tumour microenvironment and develop a potential immunotherapy strategy for BC patients.

## Methods

### Datasets

The somatic gene mutations and clinical data of 412 samples from BC patients in the United States and 101 samples from BC patients in China were downloaded from The Cancer Genome Atlas (TCGA) and International Cancer Genome Consortium (ICGC) online platforms, respectively. Tumor immune estimation resource downloaded from TIMER2.0 (http://timer.cistrome.org/).

### Data analysis

All statistical analyses were performed using Stata software 14.0 and GraphPad Prism 8.0 (San Diego, CA, USA); *P* < 0.05 was defined as statistically significant. Kaplan-Meier survival analysis was conducted to generate survival curves, which were evaluated using the log-rank test. Cox regression analyses were performed for assessing the associations of survival with clinical characteristics and *KDM6A* expression levels. The Kruskal-Wallis test was used to analyse the correlation between *KDM6A* mutation status and overall mutation counts.

CIBERSORT was conducted to evaluate the proportions of 22 tumor-infiltrating immune cells (TIICs) subsets in 412 samples from BC patients in the United States, and estimated the relative abundance of TIICs with different *KDM6A*status.

Gene set enrichment analysis (GSEA) was performed using GSEA 4.0. Student’s *t*-test was used to compare the expression level of *KDM6A* mRNA according to the *KDM6A* mutation status. The χ^2^ test was used to evaluate the association between *KDM6A* mutation status and clinical parameters. In GSEA, *P* < 0.05 and q < 0.25 were considered statistically significant.

## Results

### *KDM6A* mutation is frequent in BC

We downloaded the data, including follow-up profiles and gene expression levels, of 411 and 103 BC tissues from the TCGA and ICGC databases, respectively. Among these samples, 107/411 (25.97%) and 25/103 (24.27%) harboured a *KDM6A* mutation (Sup. 1A–C).

Missense and truncating mutations were the main mutation types spanning the entire gene (Sup.2A-B), with the former mutation type being most frequent (Sup. 3A); single nucleotide polymorphism was more common than deletion or insertion (Sup. 3B), and C > T was the most common single nucleotide variant in BC (Sup. 3C). The number of variant bases in each sample was counted and the mutation types are shown in a box plot in different colours in Sup. 3D and 3E.

### *KDM6A* mutation is associated with higher mutation counts

**Fig. 1:**
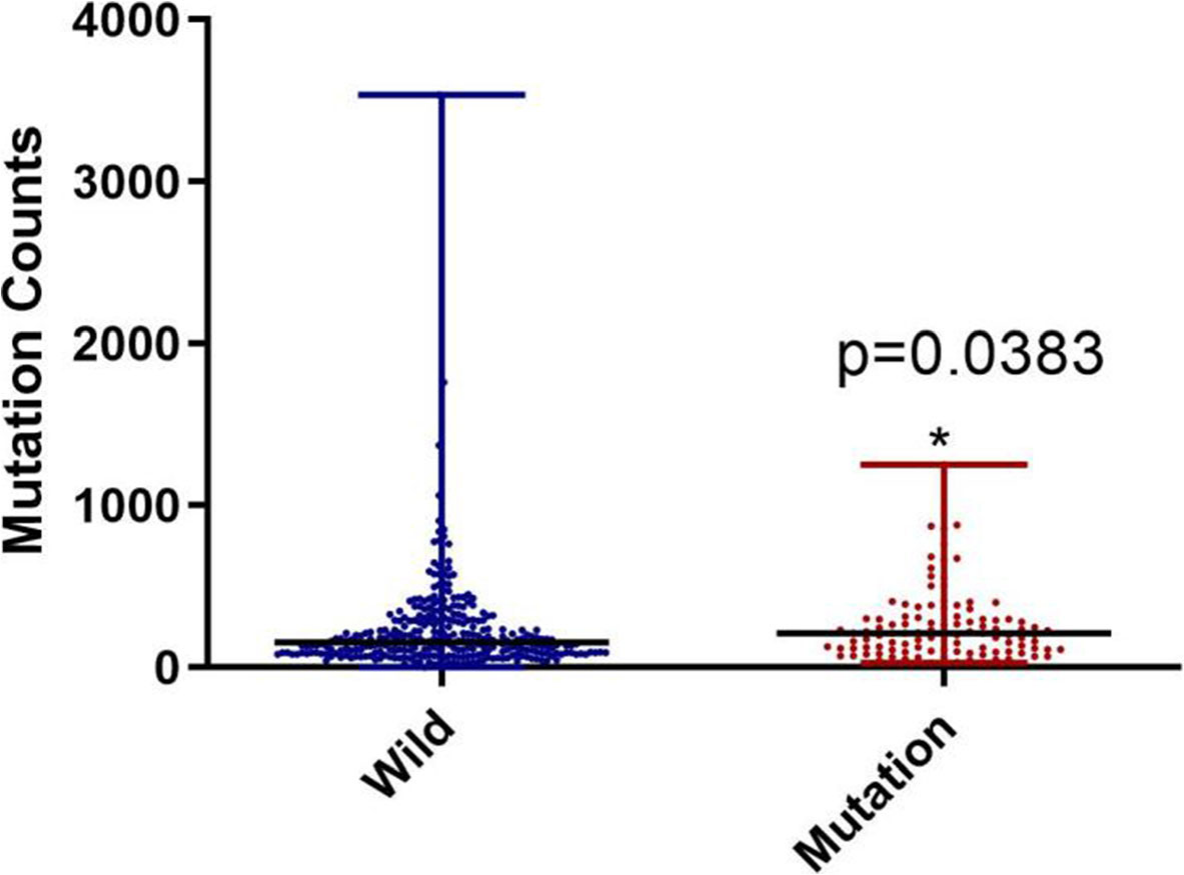
*KDM6A* mutation was associated with mutation counts. *KDM6A* mutation was associated with a higher mutation counts

BC samples with *KDM6A* mutations had higher overall mutation counts than wild-type samples in the TCGA cohort (median mutation counts: 210 vs 156.5; *p =* 0.0383, Kruskal-Wallis equality-of-populations rank test) (Fig. 1).

### *KDM6A* mutation is associated with reduced *KDM6A* mRNA levels and poor prognosis in BC

**Fig. 2:**
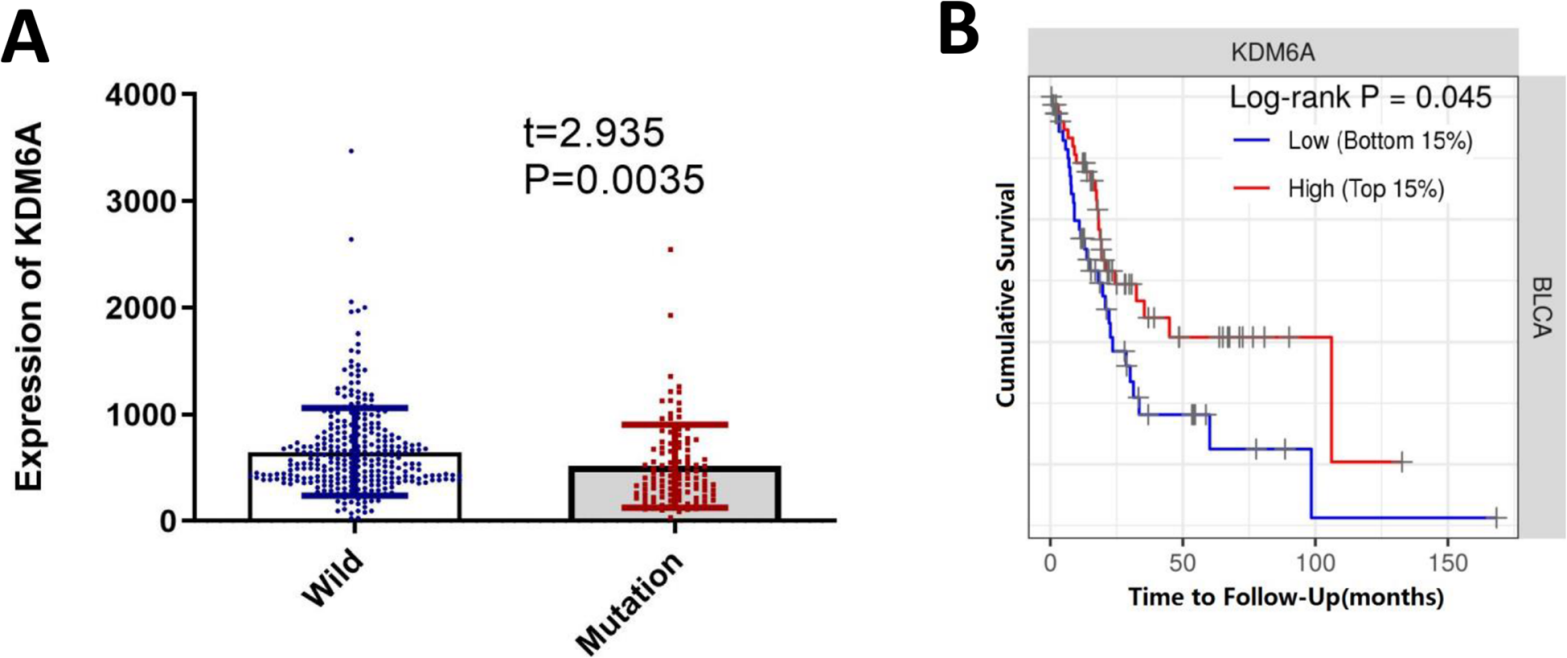
*KDM6A* mutation and bladder cancer prognosis. **A**The difference of mRNA expression between KDM6A mutation and wild. **B** Kaplan–Meier survival curves for bladder cancer patients stratified by *KDM6A* mRNA levels (Low *n* = 61, High *n* = 61)

Patients with *KDM6A* mutations had lower *KDM6A* mRNA levels than those with wild-type *KDM6A* (Fig. 2A). As shown in Sup.4, patients with *KDM6A* mutations had a higher M stage (TNM staging) than those with wild-type *KDM6A*. However, no correlation was observed between *KDM6A* mutation and age, histologic grade, or the tumour-node-metastasis (TNM) stage. Kaplan-Meier analysis showed that BC patients with a high *KDM6A* mRNA (top 15%, *n* = 61) levels had significantly longer overall survival than those with low *KDM6A* levels (bottom 15%, *n* = 61) (Fig. 2B). However, univariate and multivariate Cox regression analyses showed that a low *KDM6A* level or presence of *KDM6A* mutation was not an independent prognostic factor.

### *KDM6A* mutation is significantly correlated with tumour-infiltrating immune cells (TIICs) in the tumour microenvironment

**Fig. 3:**
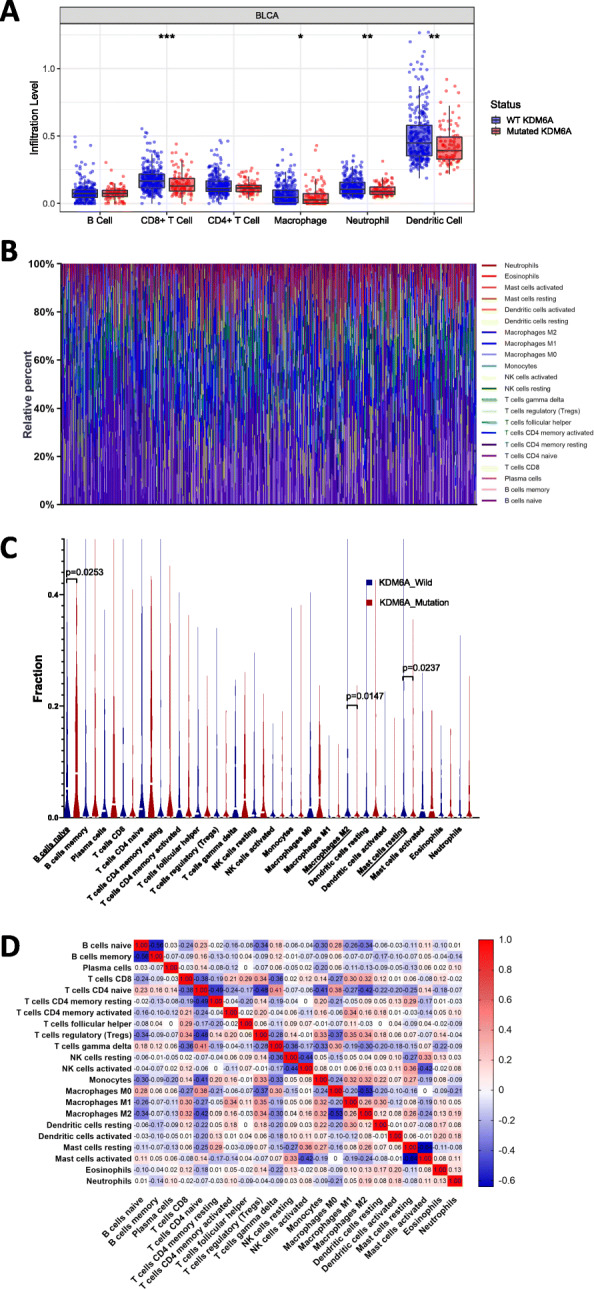
*KDM6A* mutation was correlated with tumor-infiltrating immune cells. **A** The diffrence of 8 immune cells between *KDM6A* mutation group and *KDM6A* wild-type group. Blue color represented *KDM6A* wild-type group, and red color represents *KDM6A* mutation group. * *p* < 0.05; ** *p* < 0.01; *** *p* < 0.001 **B** Stacked bar chart showed distribution of 22 immune cells in each sample. **C** Violin plot displayed the differentially infiltrated immune cells between *KDM6A* mutation group and *KDM6A* wild-type group. Blue color represented *KDM6A* wild-type group, and red color represented *KDM6A* mutation group. **D** Correlation matrix of immune cell proportions. The red color represented positive correlation and the blue color represented negative correlation

Tumor Immune Estimation Resource (TIMER) analysis showed that *KDM6A* mutation was associated with a lower number of TIICs. The infiltration levels of macrophages (*p* = 1.44e-2), CD8+ T cells (*p* = 5.79e-4), neutrophils (*p* = 4.40e-3), and resting dendritic cells (*p* = 3.70e-3) in the *KDM6A* mutation group were lower than those in the *KDM6A* wild-type group (Fig. 3A). The association between *KDM6A* mutation and different types of immune cells was significant, including dendritic cells (*p* = 9.70e-3, cor = − 0.1272), CD8 T cells (*p* = 6.0e-4, cor = − 0.1689), macrophages (*p* = 1.03e-2, cor = − 0.1262), and neutrophils (*p* = 5.1e-3, cor = − 0.1375).

To further assess the correlation of *KDM6A* mutation with TIICs in the BC microenvironment, the CIBERSORT algorithm was used. The composition of 22 TIICs in each sample varied significantly (Fig. 3B). Moreover, naive B cells were more enriched in the *KDM6A* mutation group, whereas M2 macrophages and resting mast cells were more enriched in the *KDM6A* wild-type group (Fig. 3C). Correlation analysis showed that memory-activated CD4+ T cells were positively correlated with CD8+ T cells, and also had a positive association with the number of M1 macrophages. In turn, resting macrophages were positively correlated with levels of activated natural killer (NK) cells. However, activated NK cells were negatively correlated with activated mast cells (Fig. 3D).

### Enrichment pathway analysis of *KDM6A* mutation

**Fig. 4:**
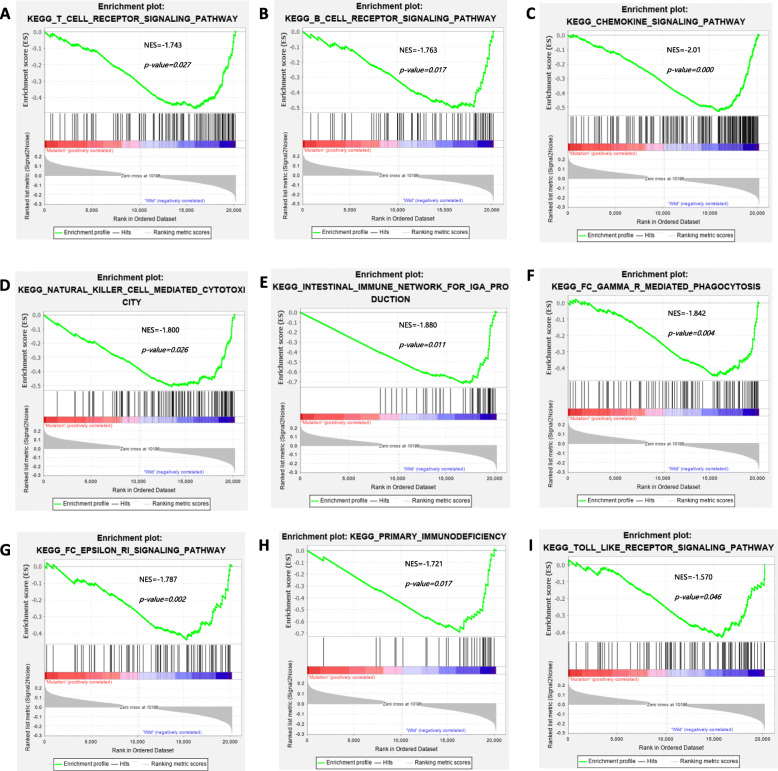
Significantly enriched pathways associated with *KDM6A* mutation. Gene set enrichment analysis has been performed with TCGA. Gene enrichment plots showed that a series of gene sets including **A** T Cell Receptor Signaling Pathway, **B** B Cell Receptor Signaling Pathway, **C** Chemokine Signaling Pathway, **D** Natural Killer Cell Mediated Cytotoxicity, **E** Intestinal Immune Network for Ig A production, **F** Fc Gamma R Mediated Phagocytosis, **G** Fc Epsilon Ri Signaling Pathway, **H** Primary Immunodeficiency and **I** Toll Like Receptor Signaling Pathway were enriched in *KDM6A* mutation group. NES, normalized enrichment score. The *p*-value is marked in each plot

We further explored the relationship between *KDM6A* mutation and the immune response. GSEA revealed that the intestinal immune network for IgA production, the chemokine signalling pathway, natural killer cell-mediated cytotoxicity, the B cell receptor signalling pathway, the T cell receptor signalling pathway, the Fc epsilon Ri signalling pathway, Fc gamma R-mediated phagocytosis, primary immunodeficiency, and the Toll-like receptor signal pathway was significantly downregulated in the *KDM6A* mutation group (Fig. 4A–I). These results implied that *KDM6A* mutation downregulates signalling pathways involved in the immune system in BC.

## Discussion

Dysfunction in demethylation occurs frequently in cancer cells, which can destroy the chromatin configuration and disrupt normal transcriptional processes. *KDM6A* is a specific H3K27me3 demethylase, and inactivating mutations in *KDM6A* have been frequently detected in BC [1]. *KDM6A* was suggested to act as a tumour suppressor in several cancers [10]. Other studies reported abnormal levels of trimethylated H3K27 (H3K27me3) in some cancers, which correlated with a poor prognosis [11–13], suggesting that demethylases are involved in both oncogenesis and tumour progression. Several recent studies identified inactivating mutations of *KDM6A* in different cancer tissues, along with decreased expression levels of *KDM6A* in cancer compared with those in normal tissues, further supporting that *KDM6A* is a tumour suppressor [14–17]. *KDM6A* mutation is common especially in women with BC [18, 19]. Mutations in or a decreased expression level of *KDM6A* was also associated with a poor prognosis of female BC patients [20].

Our present analysis of the somatic mutation landscape data of BC samples from the TCGA dataset (412 cases) and ICGC dataset (101 cases) confirmed that *KDM6A* was frequently mutated in both cohorts, in line with previous studies [6, 19]. Moreover, *KDM6A* mutation was correlated with higher overall gene mutation counts, but was not associated with clinical prognosis.

Importantly, we identified that *KDM6A* mutation in BC was negatively associated with signalling pathways involved in the immune response. We observed reduced infiltration of immune cells in *KDM6A*-mutated tissues, including neutrophils, macrophages, and CD8+ T cells, which is consistent with previous reports showing that these immune cells play key roles in the tumour microenvironment and suppress the immune response. A reduced number of CD8+ T cells in the tumour indicated a worse prognosis for patients in a previous study [21]. GSEA of Kyoto Encyclopedia of Genes and Genomes pathways between gene sets of BC samples with mutated and wild-type *KDM6A* demonstrated that the signalling pathways of cell-adhesion molecules, ECM receptor interaction, and focal adhesion were also suppressed by *KDM6A* mutation. These findings support the need for further in-depth study of the possible role of *KDM6A* in regulation of cell adhesion and morphology in BC [22]. *KDM6A* deficiency has been suggested to activate pathways of chemokines and cytokines, increase M2 macrophage polarization, increase cancer stem cell abundance, and act synergistically with p53 haploinsufficiency [23]. Given the key function of KDM6A in regulating CD4+ T cells [24], concluded that *KDM6A* likely regulates multiple immune response genes in autoimmune disease susceptibility.

There are several limitations in this study. Fist, specimens of superficial bladder cancer are a few. Therefore, more studies could conduct on invasive bladder cancer and superficial bladder cancer, separately. Second, the molecular mechanism by which *KDM6A* might suppress tumour progression need further research. Third, although our results suggested that BC patients with a high KDM6A mRNA (top 15%, *n*= 61) levels had significantly longer overall survival than those with low KDM6A levels (bottom 15%, *n* = 61), but there is insufficient high-quality data and more studies are needed. Finally, although our results showed naive B cells were more enriched in the KDM6A mutation group, it is still unclear whether naive B cells are involved in immune escape and the mechanism, further study is needed.

## Conclusions

This study identified that *KDM6A* was frequently mutated in BC, and *KDM6A* mutation was correlated with a higher overall gene mutation status. Furthermore, *KDM6A* mutation was shown to negatively regulate the signalling pathways of the immune system and to suppress antitumor immunity. These findings suggest *KDM6A* as a latent gene whose mutation could be considered as a predictive biomarker for the immune response, thereby facilitating the development of new strategies for immunotherapy and treatment monitoring in BC patients.

## Supplementary Information

**Additional file 1.**

## Data Availability

The datasets used during the present study are available from the Corresponding author upon reasonable request. Data were obtained from The Cancer Genome Atlas (TCGA; https://portal.gdc.cancer.gov), International Cancer Genome Consortium (ICGC; https://dcc.icgc.org/) and TIMER (http://timer.cistrome.org/).

## Abbreviations

*KDM6A*: Lysine-specific demethylase 6A
BC: Bladder cancer
TCGA: The Cancer Genome Atlas
ICGC: International Cancer Genome Consortium
GSEA: Gene Set Enrichment Analysis
TNM: Tumour-node-metastasis
TIICs: Tumour-infiltrating immune cells
TIMER: Tumor immune estimation resource.
*TP53*: Tumor protein p53
*CSMD3*: CUB And Sushi Multiple Domains 3
*MUC16*: Mucin 16
*STAG2*: Stromal antigen2
*PIK3CA*: Phosphoinositide-3-kinase, catalytic, alpha
*ARID1A*: AT-rich interactive domain containing protein 1A
*RB1*: Retinoblastoma1
*EP300*: E1A binding protein p300
*ERBB2*: Erb-b2 receptor tyrosine kinase 2
*ERBB3*: Erb-b2 receptor tyrosine kinase 2
*FGFR3*: Fibroblast growth factor receptor 3
NK cells: Natural killer cells

## Declarations

### Competing of interests

None declared.
