# Supplementary material for: Significance of KDM6A mutation in bladder cancer immune escape
Source: BMC Cancer. 2021 May 29;21:635. doi: 10.1186/s12885-021-08372-9 (PMC8164329; doi:10.1186/s12885-021-08372-9)
Supplement: Supplementary file 1 — Additional file 1. [file 12885_2021_8372_MOESM1_ESM.pptx]

## Slide 1
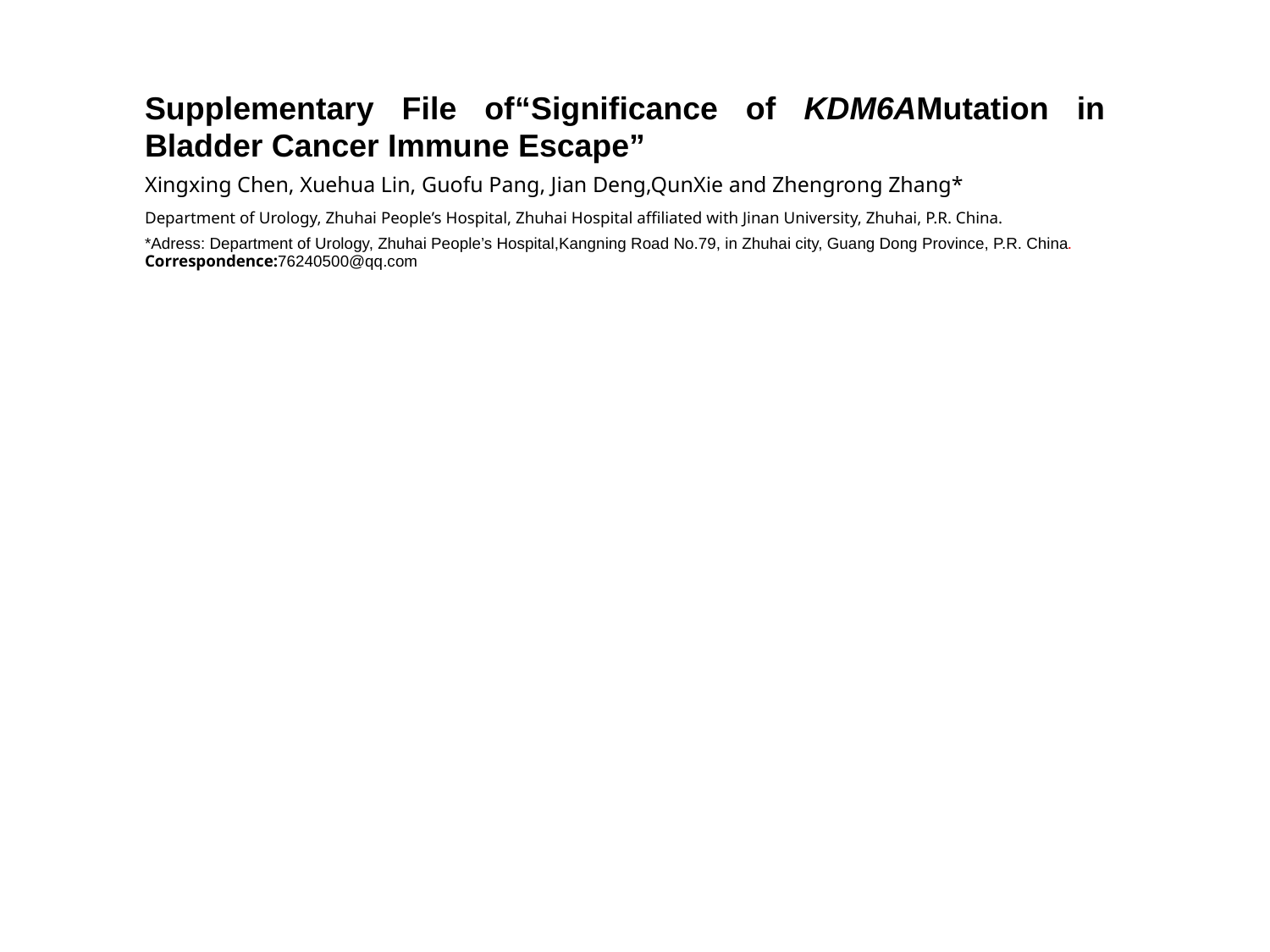

| Supplementary File of“Significance of KDM6AMutation in Bladder Cancer Immune Escape” Xingxing Chen, Xuehua Lin, Guofu Pang, Jian Deng,QunXie and Zhengrong Zhang\* Department of Urology, Zhuhai People’s Hospital, Zhuhai Hospital affiliated with Jinan University, Zhuhai, P.R. China. \*Adress: Department of Urology, Zhuhai People’s Hospital,Kangning Road No.79, in Zhuhai city, Guang Dong Province, P.R. China. Correspondence:76240500@qq.com |
| --- |

## Slide 2
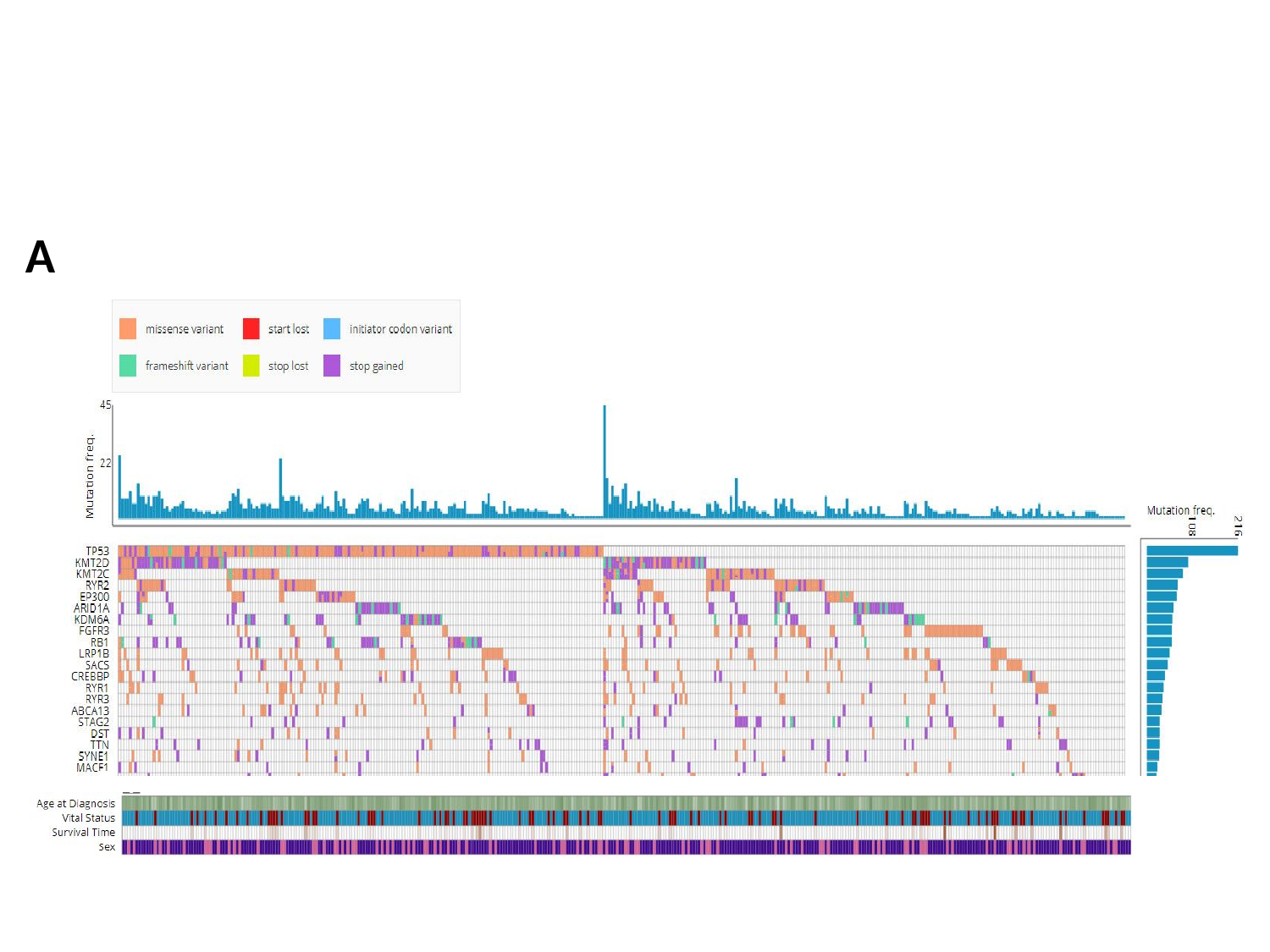

A

## Slide 3
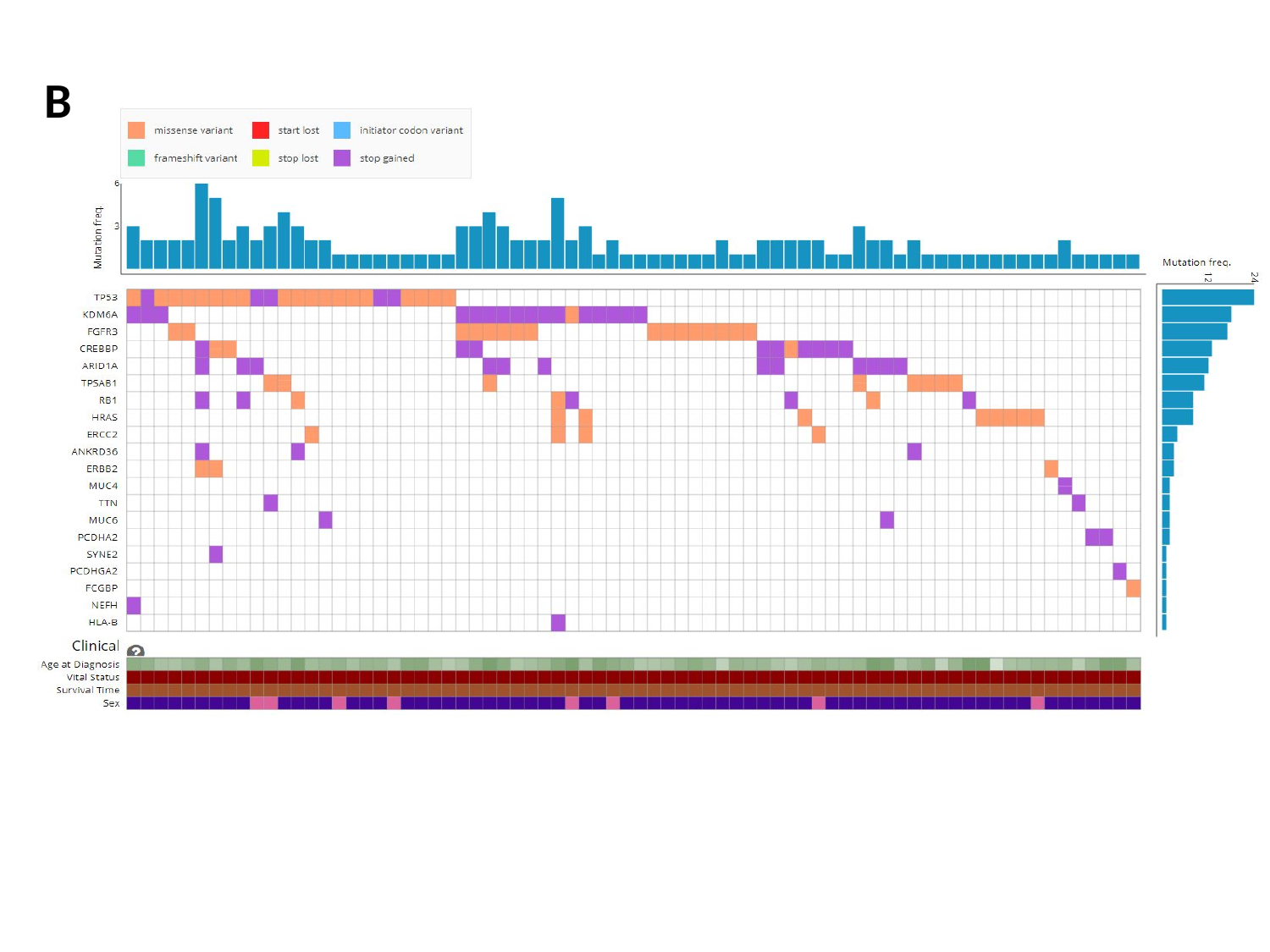

B

## Slide 4
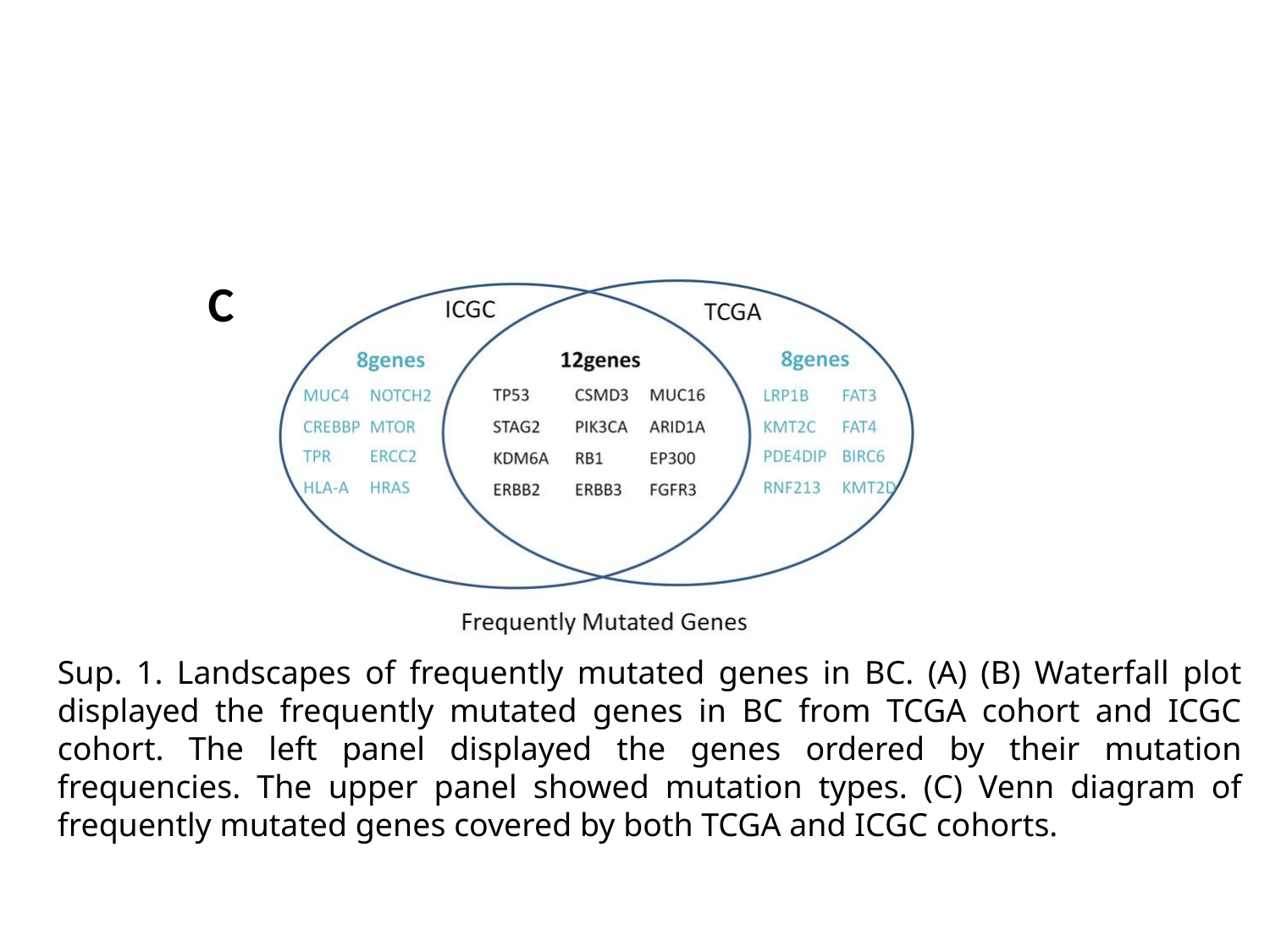

C
Sup. 1. Landscapes of frequently mutated genes in BC. (A) (B) Waterfall plot displayed the frequently mutated genes in BC from TCGA cohort and ICGC cohort. The left panel displayed the genes ordered by their mutation frequencies. The upper panel showed mutation types. (C) Venn diagram of frequently mutated genes covered by both TCGA and ICGC cohorts.

## Slide 5
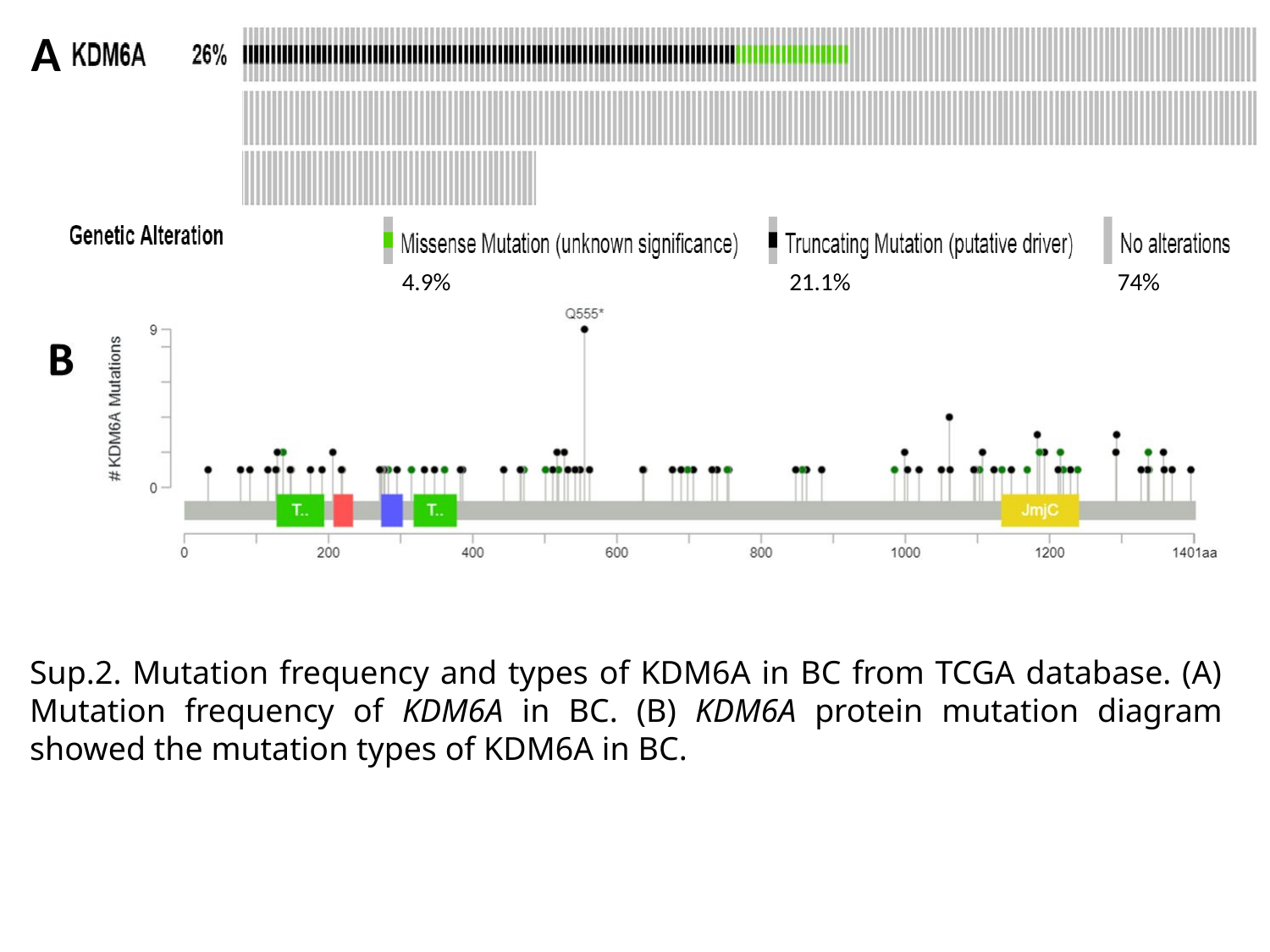

A
4.9%
74%
21.1%
Sup.2. Mutation frequency and types of KDM6A in BC from TCGA database. (A) Mutation frequency of KDM6A in BC. (B) KDM6A protein mutation diagram showed the mutation types of KDM6A in BC.

## Slide 6
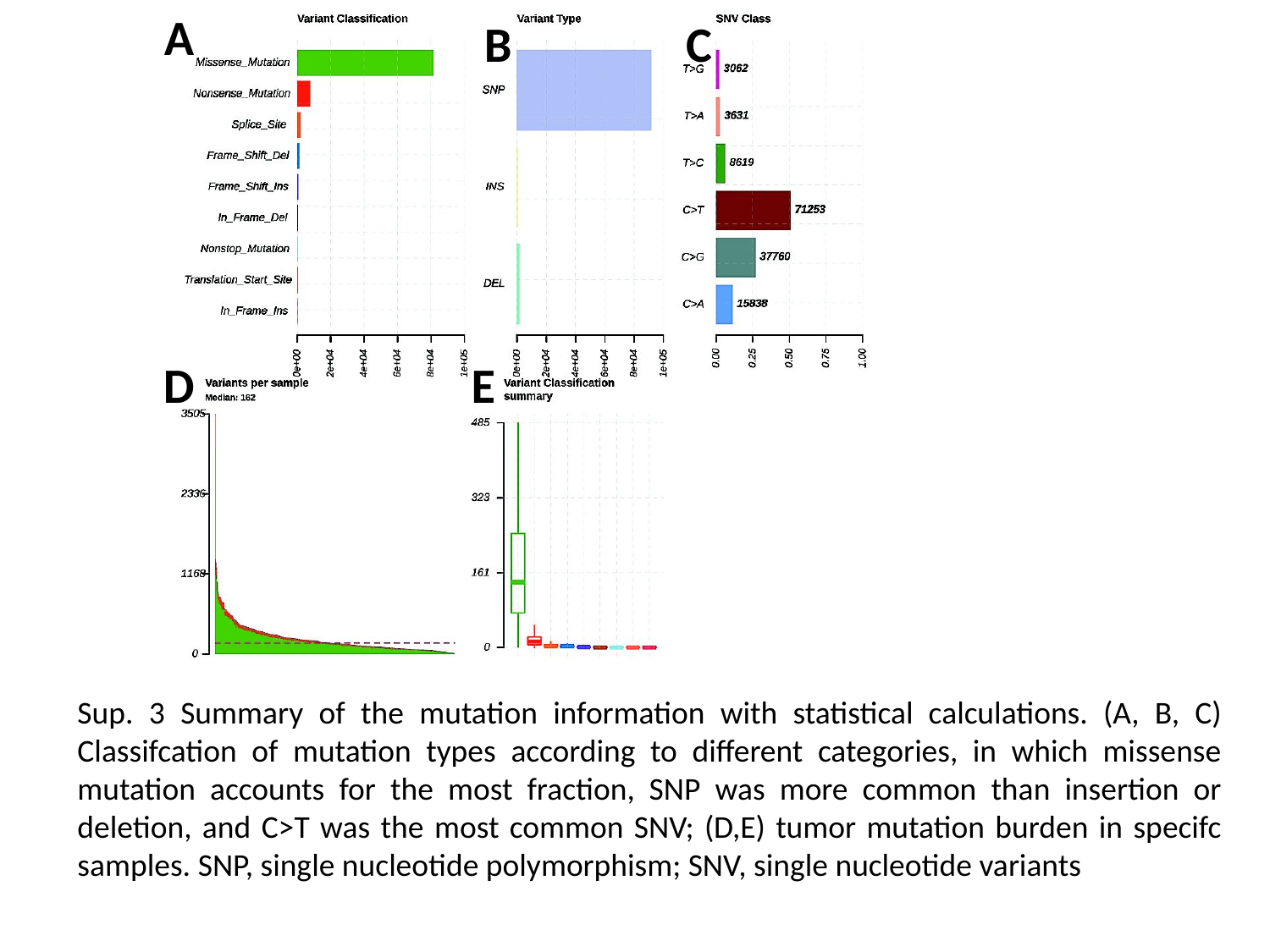

A
B
C
D
E
Sup. 3 Summary of the mutation information with statistical calculations. (A, B, C) Classifcation of mutation types according to different categories, in which missense mutation accounts for the most fraction, SNP was more common than insertion or deletion, and C>T was the most common SNV; (D,E) tumor mutation burden in specifc samples. SNP, single nucleotide polymorphism; SNV, single nucleotide variants

## Slide 7
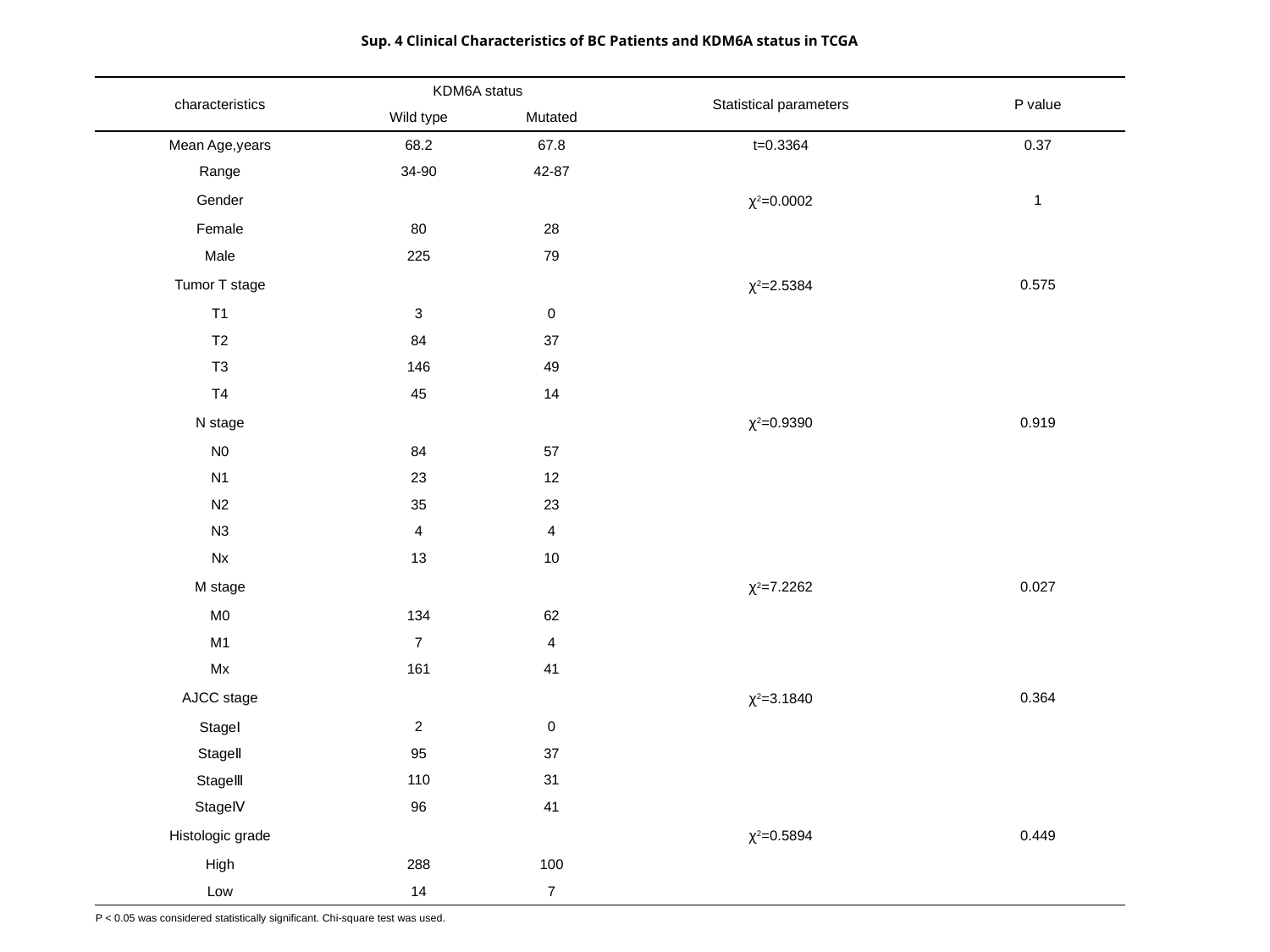

| Sup. 4 Clinical Characteristics of BC Patients and KDM6A status in TCGA | | | | |
| --- | --- | --- | --- | --- |
| characteristics | KDM6A status | | Statistical parameters | P value |
| | Wild type | Mutated | | |
| Mean Age,years | 68.2 | 67.8 | t=0.3364 | 0.37 |
| Range | 34-90 | 42-87 | | |
| Gender | | | χ2=0.0002 | 1 |
| Female | 80 | 28 | | |
| Male | 225 | 79 | | |
| Tumor T stage | | | χ2=2.5384 | 0.575 |
| T1 | 3 | 0 | | |
| T2 | 84 | 37 | | |
| T3 | 146 | 49 | | |
| T4 | 45 | 14 | | |
| N stage | | | χ2=0.9390 | 0.919 |
| N0 | 84 | 57 | | |
| N1 | 23 | 12 | | |
| N2 | 35 | 23 | | |
| N3 | 4 | 4 | | |
| Nx | 13 | 10 | | |
| M stage | | | χ2=7.2262 | 0.027 |
| M0 | 134 | 62 | | |
| M1 | 7 | 4 | | |
| Mx | 161 | 41 | | |
| AJCC stage | | | χ2=3.1840 | 0.364 |
| StageⅠ | 2 | 0 | | |
| StageⅡ | 95 | 37 | | |
| StageⅢ | 110 | 31 | | |
| StageⅣ | 96 | 41 | | |
| Histologic grade | | | χ2=0.5894 | 0.449 |
| High | 288 | 100 | | |
| Low | 14 | 7 | | |
| P < 0.05 was considered statistically significant. Chi-square test was used. | | | | |
